# Alterations in gut microbiota among maintenance hemodialysis patients with CKD-associated pruritus: a cross-sectional comparative study of two cohorts

**DOI:** 10.3389/fcimb.2026.1917432

**Published:** 2026-08-28

**Authors:** Min Huang, Saiping Chen, Yuqing Wang, Jiarui Wang, Junyi Liu, Mengqi Wu, Bin Lu, Aiping Zhang, YanQin Zhu, Fenggui Zhu, Hong Liu, Xueyan Zeng, Shilei Chen, Xin Zhou, Riyang Lin

**Affiliations:** 1 Department of Nephrology, Hangzhou Traditional Chinese Medicine Hospital of Zhejiang Chinese Medical University, Hangzhou, Zhejiang, China; 2 Xingqiao Street Community Health Service Center, Hangzhou, Zhejiang, China; 3 Department of Nephrology, Sichuan Second Hospital of Traditional Chinese Medicine, Chengdu, Sichuan, China; 4 Department of General Medicine, Hangzhou Xihu District Zhuantang Street Community Health Service Centre, Hangzhou, Zhejiang, China; 5 Tianshui Wulin Street Community Health Care Centre, Hangzhou, Zhejiang, China; 6 Key Laboratory of Kidney Disease Prevention and Control Technology, Hangzhou, China

**Keywords:** 16S rRNA gene sequencing, chronic kidney disease-associated pruritus, cross-sectional study, gut microbiota, maintenance hemodialysis, parasutterella

## Abstract

**Objective:**

Chronic kidney disease-associated pruritus (CKD-aP) is a common and burdensome complication in patients receiving maintenance hemodialysis (MHD), but its pathogenesis remains incompletely understood. This study aimed to characterize gut microbial differences between MHD patients with active CKD-aP and those without active pruritus.

**Methods:**

In this single-center cross-sectional study, 89 patients receiving MHD were enrolled, including 45 with active CKD-aP (HDUP) and 44 without active pruritus (HDNUP). Fecal samples were analyzed using 16S rRNA gene sequencing. Microbial diversity, taxonomic composition, differentially abundant taxa, and predicted functional profiles were compared between groups. Multivariable association analysis was performed using MaAsLin2 at the phylum, class, order, family, genus, and species levels, with adjustment for age, sex, post-dialysis dry weight, dialysis vintage, the presence of diabetes mellitus, and a documented history of hyperphosphataemia.

**Results:**

Alpha-diversity indices did not differ significantly between groups. Bray–Curtis-based analysis of similarities showed no difference in overall community structure (R = −0.00463, p = 0.5882), whereas unweighted UniFrac-based analysis detected a statistically significant but very small difference (R = 0.03449, p = 0.0183). Fourteen genera showed nominal differences in univariable analyses. After multivariable adjustment and false-discovery-rate (FDR) correction, significant associations were identified only at the family and order levels. *Parasutterella* showed a nominal genus-level association but did not remain statistically significant after FDR correction. LEfSe identified 31 candidate differentially enriched taxonomic features. PICRUSt2 analysis suggested nominal differences in six predicted pathways, including higher predicted protein digestion and absorption in HDNUP and higher predicted RIG-I-like receptor signaling and ether lipid metabolism in HDUP.

**Conclusion:**

Active CKD-aP was associated with selective differences in gut microbial relative abundance rather than broad alterations in microbial richness, diversity, or dominant community structure. *Parasutterella* showed a nominal genus-level association with the absence of active pruritus, although this association did not remain statistically significant after FDR correction. Other taxonomic and predicted functional findings were exploratory and require validation in longitudinal, multicenter studies incorporating absolute microbial quantification and metabolomic analyses.

## 1 Introduction

Kidney failure requiring maintenance dialysis represents a growing global public health burden (Levey et al., 2020; Thurlow et al., 2021). Population aging and the increasing prevalence of chronic conditions such as diabetes and hypertension have contributed to a continuing rise in the number of people living with kidney failure worldwide (Kovesdy, 2022). Maintenance hemodialysis (MHD) is one of the principal kidney replacement therapies and substantially prolongs patient survival (Thurlow et al., 2021). Nevertheless, patients receiving MHD frequently experience persistent and burdensome symptoms, among which pruritus is one of the most common and distressing (Pisoni et al., 2006). Uremic pruritus, currently referred to as chronic kidney disease-associated pruritus (CKD-aP), is a debilitating complication affecting patients with advanced CKD, particularly those undergoing dialysis (Rayner et al., 2017; Osakwe and Rout, 2026). Approximately 31%–40% of patients receiving MHD experience moderate-to-severe pruritus, which is frequently associated with sleep disturbance, psychological distress, impaired quality of life, and adverse clinical outcomes (Pisoni et al., 2006; Kimata et al., 2014).

The pathogenesis of CKD-aP is complex and remains incompletely understood. Rather than being attributable to a single factor, CKD-aP is thought to result from interactions among multiple abnormalities associated with the uremic milieu, including chronic systemic and cutaneous inflammation, immune dysregulation, impaired epidermal barrier function, altered peripheral or central neural signaling, and the accumulation of uremic solutes (Azimi and Lerner, 2024; Rigatto et al., 2024; Zhai et al., 2024; Zibandeh et al., 2024). These mechanisms may interact and contribute to the considerable heterogeneity in the presence and severity of pruritus among patients with otherwise similar dialysis characteristics. Moreover, persistent pruritus remains common despite improvements in dialysis adequacy and conventional clinical management, indicating that established dialysis-related and biochemical factors do not fully explain CKD-aP (Rigatto et al., 2024; Skrzypczak et al., 2024). Importantly, although uremic solute accumulation has long been proposed as a possible contributor to CKD-aP, previous work has not identified a specific uremic solute that is consistently associated with itch. EUTox-related work has emphasized the complexity of uremic toxicity and the limitations of attributing the uremic syndrome to individual retained solutes (Vanholder et al., 2008). Consistent with this, Bolanos et al. found no individual plasma solute or group of solutes associated with pruritus in patients receiving hemodialysis (Bolanos et al., 2021). Therefore, potential microbiota-related mechanisms in CKD-aP should be interpreted cautiously and should not be assumed to operate through a specific gut-derived uremic solute pathway.

The gut microbiota has emerged as a potential contributor to systemic complications in patients receiving hemodialysis. In patients with kidney failure, the uremic milieu, dietary restrictions, altered intestinal motility, constipation, and medication exposure may disrupt both the composition and metabolic activity of the intestinal microbiota (Luo et al., 2021; Du et al., 2024; Lazarevic et al., 2024). These alterations may impair intestinal barrier integrity, facilitate the translocation of bacterial components and microbiota-derived metabolites, and promote systemic inflammation and immune activation. The gut microbiota may influence intestinal barrier function, systemic inflammatory and immune signaling, and the generation of a wide range of microbial metabolites. However, current evidence has not established a specific microbiota-derived uremic solute as a mediator of CKD-aP.

Direct human evidence linking the gut microbiota to CKD-aP remains limited. Most microbiome studies in CKD have compared patients with healthy individuals or focused on renal function, nutritional status, inflammation, and cardiovascular outcomes rather than pruritus as a predefined phenotype. Ko et al. recently compared the fecal microbiota of hemodialysis patients with and without uremic pruritus and found no significant difference in alpha diversity but identified differences in beta diversity and several candidate taxa associated with pruritus status (Ko et al., 2025). However, gut microbial profiles are strongly influenced by geographic and dietary backgrounds, bowel habits, dialysis duration, medication exposure, comorbidities, sequencing platforms, and bioinformatics methods. Independent studies involving clinically comparable groups and covariate-adjusted analyses are therefore needed. It also remains unclear whether CKD-aP is associated with broad restructuring of the intestinal microbial community or with more selective differences involving particular taxa and predicted microbial functions.

Accordingly, this single-center, cross-sectional exploratory study used 16S rRNA gene sequencing to compare the fecal microbiota of MHD patients with active CKD-aP and those without active pruritus. We assessed microbial richness and diversity, overall community structure, taxonomic composition, differentially abundant taxa, and predicted functional profiles. Exploratory multivariable association analyses were performed using MaAsLin2 across six taxonomic levels, with adjustment for selected demographic and clinical covariates, including age, sex, post-dialysis dry weight, dialysis vintage, the presence of diabetes mellitus, and a documented history of hyperphosphataemia. This study aimed to identify candidate gut microbial features associated with pruritus status and to generate hypotheses for subsequent longitudinal, multicenter, and multi-omics investigations.

## 2 Materials and methods

### 2.1 Study design and ethical approval

This single-center, cross-sectional exploratory study was conducted at the Hemodialysis Center of Hangzhou Traditional Chinese Medicine Hospital of Zhejiang Chinese Medical University, Hangzhou, China. Recruitment, clinical assessment, and fecal sample collection were conducted between April and July 2025. The study protocol was approved by the Ethics Committee of Hangzhou Traditional Chinese Medicine Hospital (approval No. 2025KLL125) and was conducted in accordance with the Declaration of Helsinki and applicable institutional requirements. All participants provided written informed consent before enrollment.

### 2.2 Study participants

Adult patients receiving maintenance hemodialysis at the study center were screened according to predefined eligibility criteria. Chronic kidney disease-associated pruritus was diagnosed using prespecified clinical criteria consistent with established definitions. CKD-aP was defined as pruritus that could not be adequately explained by another clinically identifiable condition, occurred on at least 3 days within a 2-week period, recurred several times per day for minutes or longer, affected daily life, and had persisted or recurred for at least 6 weeks. Clinically identifiable dermatologic, hepatobiliary, endocrine, infectious, parasitic, and drug-related causes of pruritus were evaluated through medical history, physical examination, medication review, and routine laboratory testing. Secondary scratch-related skin lesions were permitted, whereas patients with primary skin disease sufficient to explain the pruritus were excluded.

At enrollment, patients satisfying the diagnostic criteria for CKD-aP with ongoing active pruritus were allocated to the maintenance hemodialysis with active CKD-aP group (HDUP). Participants without active pruritus at enrollment were assigned to the maintenance hemodialysis without active pruritus group (HDNUP). A total of 89 participants were included in microbiome sequencing and the final analysis, including 45 participants in the HDUP group and 44 in the HDNUP group.

The inclusion criteria were: (1) age ≥18 years; (2) a diagnosis of kidney failure and receipt of MHD for at least 3 consecutive months; (3) a clinically stable condition with no change in the dialysis prescription during the preceding month; (4) the ability to understand and complete the required clinical assessments and provide a fecal sample and (5) provision of written informed consent.

The exclusion criteria were: (1) acute infection, fever, or acute gastrointestinal or respiratory illness within the preceding month; (2) use of antibiotics, probiotics, prebiotics, or proton pump inhibitors within the preceding month; (3) organic gastrointestinal disease, including inflammatory bowel disease, intestinal obstruction, gastrointestinal malignancy, or a history of major gastrointestinal surgery; (4) a clinically identifiable alternative cause of pruritus, including a primary dermatologic disorder, cholestatic liver disease, thyroid dysfunction, parasitic infestation, or suspected drug-induced pruritus; (5) severe or clinically unstable systemic disease that could interfere with study participation or fecal sample collection, including decompensated heart failure, severe hepatic dysfunction, or active malignancy; and (6) incomplete clinical information, failure to complete the required assessments, or failure to provide a qualified fecal sample.

**Figure 1:**
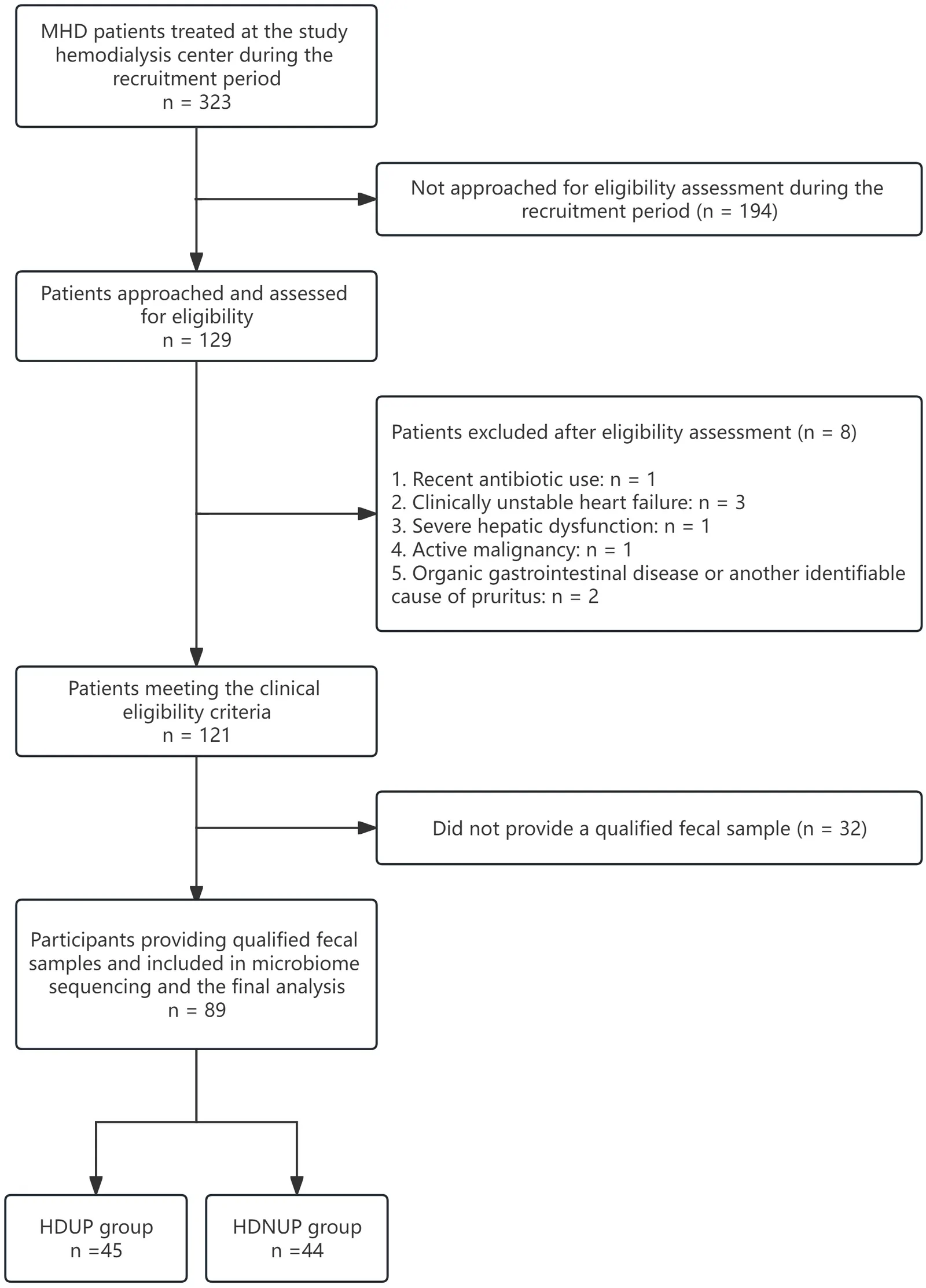
Flowchart of participant screening, fecal sample collection, and inclusion in the microbiome analysis. MHD, maintenance hemodialysis; CKD-aP, chronic kidney disease-associated pruritus; HDUP, maintenance hemodialysis patients with active CKD-aP; HDNUP, maintenance hemodialysis patients without active pruritus.

During the recruitment period, 323 patients were receiving maintenance hemodialysis at the study center. Among them, 129 patients were approached and assessed for eligibility. Eight patients were excluded after eligibility assessment because of recent antibiotic use (n = 1), clinically unstable heart failure (n = 3), severe hepatic dysfunction (n = 1), active malignancy (n = 1), or organic gastrointestinal disease or another identifiable cause of pruritus (n = 2). Of the remaining 121 patients who met the clinical eligibility criteria, 32 did not provide a qualified fecal sample. Finally, 89 participants were included in the microbiome sequencing and the final analysis, including 45 participants in the HDUP group and 44 in the HDNUP group (**Figure 1**).

### 2.3 Collection of clinical basic data

Demographic and clinical data were collected via standardized interviews and electronic medical records, including age, sex, body mass index (BMI), post-dialysis body weight, dialysis vintage, dialysis frequency and modality, dialyzer type, vascular access, primary renal disease, and relevant comorbidities. Laboratory indices measured within the time window closest to stool sampling covered white blood cell count, platelet count, serum calcium, serum phosphorus, intact parathyroid hormone (iPTH), β2-microglobulin, C-reactive protein (CRP), serum albumin, and dialysis adequacy (Kt/V). Medication history over the prior 3 months, especially sevelamer and/or cinacalcet, was also documented. Documented histories of hyperphosphataemia and secondary hyperparathyroidism were extracted from the electronic medical records. The serum phosphate and intact parathyroid hormone values reported in **Table 1** were single measurements obtained closest to fecal sample collection and were not used to determine or reclassify the documented diagnostic status.

**Table 1:** Demographic and clinical characteristics of the study population (n = 89) .

Variable	HDNUP		HDUP		P
Number of participants	44		45		
Age (years)	66.00	(43-84)	67.00	(36-85)	0.405
Sex (male/female)	24/20		26/19		0.759
Dialysis duration (years)	3.00	(1-19)	5.00	(0.6-44)	0.203
Post-dialysis weight (kg)	55.250	(36.5-87.0)	57.100	(41.2-76.1)	0.655
Kt/V (Daugirdas formula)	1.49	(1.09-2.24)	1.47	(0.92-2.01)	0.279
Serum calcium (mmol/L)	2.21 ± 0.22		2.27 ± 0.17		0.152
Serum phosphorus (mmol/L)	1.66	(0.91-2.65)	1.69	(0.80-4.35)	0.263
Parathyroid hormone (PTH, pg/mL)	291.03	(2.14-1913.25)	265.29	(17.73-1135.98)	0.726
β2-microglobulin (mg/L)	28.27 ± 6.83		28.78 ± 6.96		0.727
C-reactive protein (CRP, mg/L)	1.6050	(0.32-21.89)	1.5600	(0.05-18.28)	0.768
Platelet count	163.86 ± 38.01		165.82 ± 52.26		0.840
White blood cell count	5.18 ± 1.35		5.59 ± 1.71		0.213
Use of sevelamer and/or cinacalcet	10	22.7%	13	28.9%	0.507
Hyperphosphatemia	20	45.5%	30	66.7%	0.044
Secondary hyperparathyroidism	20	45.5%	29	64.4%	0.072
Hypertension	41	93.2%	42	93.3%	1.000
Diabetes mellitus	17	38.6%	14	31.1%	0.456
BMI	21.93	(14.81-30.10)	21.35	(16.81-27.29)	0.728

Continuous variables are presented as mean ± standard deviation or median (range), as appropriate, and categorical variables as n (%).

HDUP, patients receiving maintenance hemodialysis with active CKD-associated pruritus; HDNUP, patients receiving maintenance hemodialysis without active pruritus; BMI, body mass index; Kt/V, dialysis adequacy index calculated using the Daugirdas formula; PTH, parathyroid hormone; CRP, C-reactive protein. P values were calculated to compare differences between HDNUP and HDUP.

### 2.4 Fecal sample collection and DNA extraction

Fresh fecal material was collected using a sterile fecal swab, transferred into a sterile collection tube, and immediately stored at −80 °C. Samples were maintained under frozen conditions and transported on dry ice to Hangzhou Cosmos Wisdom Company (Hangzhou, China) for DNA extraction, library preparation, sequencing, and bioinformatics processing.

### 2.5 16S rRNA gene targeted amplification and sequencing

Total microbial genomic DNA was extracted from fecal samples using the FastPure Stool DNA Isolation Kit according to the manufacturer’s instructions. DNA integrity was evaluated by 1% agarose gel electrophoresis, and DNA concentration and purity were measured using a NanoDrop 2000 spectrophotometer. The V3–V4 hypervariable region of the bacterial 16S rRNA gene was amplified using barcode-tagged primers 338F (5′-ACTCCTACGGGAGGCAGCAG-3′) and 806R (5′-GGACTACHVGGGTWTCTAAT-3′). PCR products were purified by 2% agarose gel electrophoresis, and the target amplicons were recovered and quantified using a Synergy HTX multimode reader. Sequencing libraries were prepared using the NEXTFLEX Rapid DNA-Seq Kit and sequenced on an Illumina NextSeq 2000 platform using a paired-end 300-bp strategy.

### 2.6 Sequence processing and microbiome analysis

Raw paired-end reads were quality-filtered using fastp and merged using FLASH. Primer sequences were identified and removed using cutadapt. The DADA2 workflow implemented in QIIME 2 was used for denoising, error correction, chimera removal, and generation of amplicon sequence variants (ASVs). Taxonomic assignment was performed using the RDP classifier against the SILVA 16S rRNA gene database (release 138) with a confidence threshold of 70%. Community composition was summarized at multiple taxonomic levels, and rarefaction curves were used to assess sequencing depth.

Alpha diversity was evaluated using the ACE, Chao1, observed-features, Shannon, and Simpson indices, and between-group differences were assessed using the Wilcoxon rank-sum test. Bray–Curtis, Jaccard, weighted UniFrac, and unweighted UniFrac distance matrices were calculated for beta-diversity analysis. Principal coordinate analysis and non-metric multidimensional scaling were used for ordination, and analysis of similarities was used to test between-group differences in overall community composition.

Between-group differences in taxon abundance were initially evaluated using the Wilcoxon rank-sum test. Linear discriminant analysis effect size (LEfSe) was used to identify differentially enriched taxa, with p < 0.05 and a linear discriminant analysis score >2.0 as the screening thresholds. Exploratory multivariable association analyses were subsequently performed using MaAsLin2 across six taxonomic levels, including phylum, class, order, family, genus, and species, with age, sex, post-dialysis dry weight, dialysis vintage, the presence of diabetes mellitus, and a documented history of hyperphosphataemia included as covariates. These variables were selected to account for relevant demographic and clinical variation while maintaining model parsimony given the sample size. Several potentially relevant dietary, gastrointestinal, dermatological, and medication-related variables were not included because they had not been collected in a sufficiently standardized and complete manner. Benjamini–Hochberg false-discovery-rate-adjusted p values were reported as q values, and q < 0.05 was considered statistically significant.

### 2.7 Functional prediction and statistical analysis

Potential microbial functions were inferred from the ASV profiles using Phylogenetic Investigation of Communities by Reconstruction of Unobserved States 2 (PICRUSt2, version 2.2.0). Predicted gene-family profiles were mapped to Kyoto Encyclopedia of Genes and Genomes pathways, and between-group pathway differences were evaluated using STAMP. Because these functions were inferred from 16S rRNA gene data rather than measured directly, pathway-level findings based on nominal p values were treated as exploratory and interpreted cautiously.

For clinical data, continuous variables were summarized as mean ± standard deviation when approximately normally distributed and as median (range) otherwise. Between-group comparisons were performed using the independent-samples t-test or Mann–Whitney U test, as appropriate. Categorical variables were presented as counts and percentages and compared using the chi-square test or Fisher’s exact test. All tests were two-sided, and p < 0.05 was considered statistically significant unless otherwise specified. No subgroup or interaction analyses were prespecified. Given the exploratory design and limited sample size, all analyses were conducted in the overall study population, and *post hoc* subgroup analyses were not performed.

## 3 Results

### 3.1 Demographic and clinical characteristics of the participants

A total of 89 patients receiving maintenance hemodialysis were included in the study, comprising 45 patients in the HDUP group and 44 patients in the HDNUP group. No statistically significant between-group differences were observed in age, body mass index, post-dialysis body weight, dialysis duration, dialysis adequacy, serum calcium, serum phosphorus, intact parathyroid hormone, beta-2 microglobulin, C-reactive protein, platelet count, or white blood cell count. No statistically significant between-group differences were observed in hypertension, diabetes mellitus, documented secondary hyperparathyroidism, or the use of sevelamer and/or cinacalcet (all p > 0.05). However, a documented history of hyperphosphataemia was more frequent in the HDUP group than in the HDNUP group (p = 0.044). In contrast, the serum phosphorus concentration measured closest to fecal sampling did not differ significantly between the groups (p = 0.263) (**Table 1**).

The documented history of hyperphosphataemia and the contemporaneous serum phosphorus measurement reflected different clinical time frames and were therefore not considered equivalent indicators of phosphate status. The distributions of primary renal diseases are presented descriptively (**Table 2**).

**Table 2:** Distribution of primary kidney diseases among the study participants (n = 89).

Primary nephropathy	HDNUP		HDUP	
Chronic glomerulonephritis	25	56.8%	23	51.1%
Diabetic nephropathy	11	25.0%	9	20.0%
IgA nephropathy	2	4.5%	7	15.6%
Polycystic kidney disease	3	6.8%	5	11.1%
Hypertensive nephropathy	1	2.3%	0	0%
Systemic lupus erythematosus	1	2.3%	0	0%
Systemic vasculitis	1	2.3%	0	0%
Renal amyloidosis	0	0%	1	2.3%

Categorical variables as n (percentage); IgA, immunoglobulin A.

### 3.2 Species diversity analysis

#### 3.2.1 α-diversity analysis

**Figure 2:**
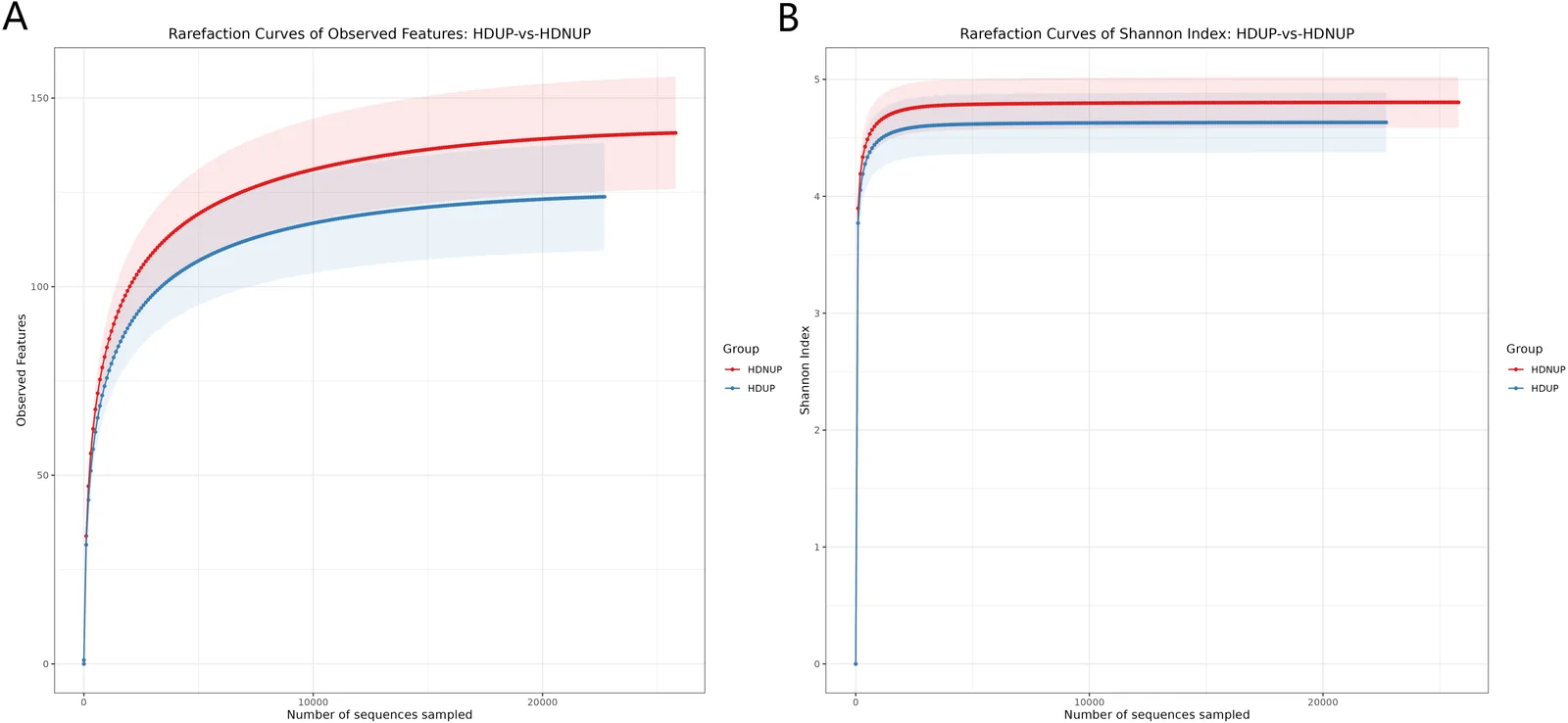
Rarefaction curves of α-diversity analysis. **(a)** Observed features curve; **(b)** Shannon index. Different colors in the figure represent samples from different groups, with the abscissa indicating the number of randomly sampled sequences and the ordinate indicating the index values.

The ACE, Chao1, observed-features, Shannon, and Simpson indices did not differ significantly between the HDUP and HDNUP groups (all p > 0.05). The rarefaction curves approached plateaus in both groups, indicating adequate sequencing depth and suggesting comparable microbial richness and diversity between the two groups (**Figure 2**).

#### 3.2.2 β-diversity analysis

β-diversity analysis showed substantial overlap in gut microbial community structure between the HDUP and HDNUP groups. Principal coordinate analysis based on Bray–Curtis distance did not reveal clear separation between the two groups, with the first two principal coordinates explaining 10.09% and 9.11% of the total variation, respectively. Similarly, non-metric multidimensional scaling based on unweighted UniFrac distance showed extensive overlap between groups. The NMDS stress value was 0.2142, indicating that the two-dimensional ordination should be interpreted cautiously.

**Figure 3:**
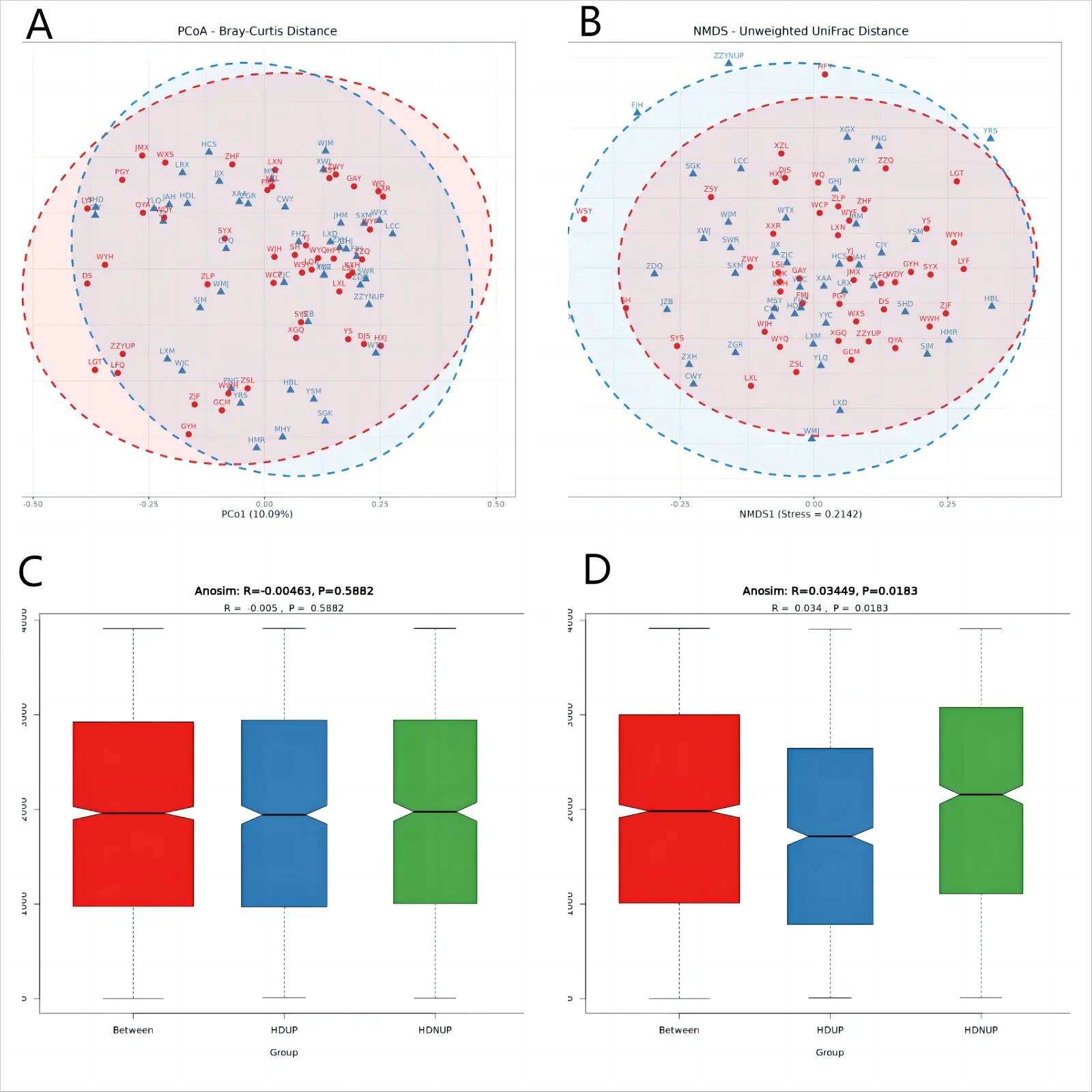
β-diversity analysis of gut microbiota between HDNUP and HDUP. **(a)** Principal Coordinate Analysis (PCoA) based on Bray-Curtis distance; **(b)** Non-metric Multidimensional Scaling (NMDS) based on Unweighted UniFrac distance; **(c)** Analysis of Similarities (ANOSIM) based on Bray-Curtis distance; **(d)** Analysis of Similarities (ANOSIM) based on Unweighted UniFrac distance. The results showed that Analysis of Similarities (ANOSIM) based on Unweighted UniFrac distance revealed a difference (p = 0.0183).

ANOSIM based on Bray–Curtis distance showed no significant difference in overall microbial community structure between the HDUP and HDNUP groups (R = −0.00463, p = 0.5882). In contrast, ANOSIM based on unweighted UniFrac distance detected a statistically significant but very small between-group difference (R = 0.03449, p = 0.0183). These findings indicate that the two groups had broadly similar overall microbial community structures, with only subtle differences in the presence or absence of phylogenetically related taxa rather than a marked restructuring of the dominant microbial communities (**Figure 3**).

### 3.3 Species difference analysis

#### 3.3.1 Overall taxonomic composition

**Figure 4:**
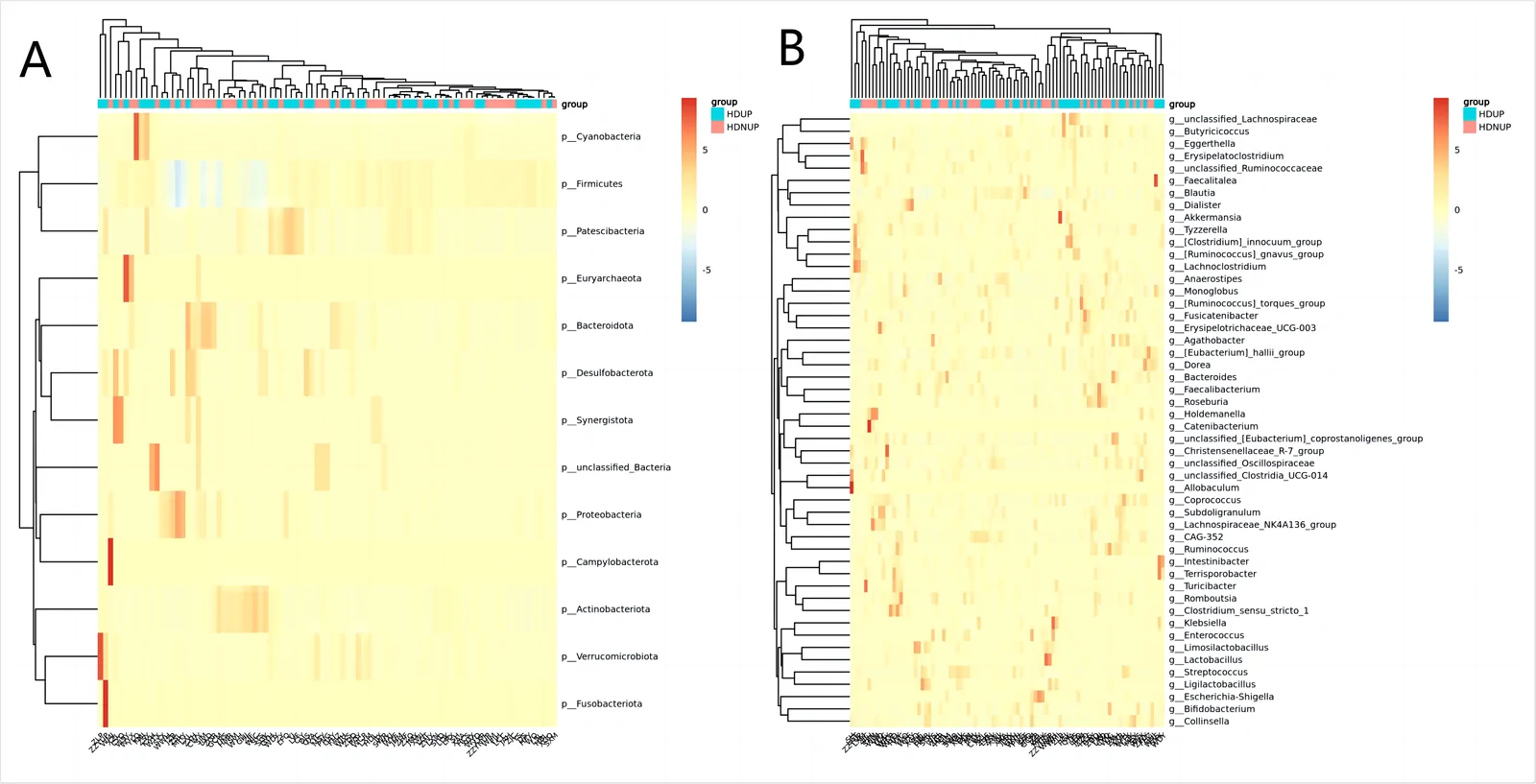
Species composition heat map at the phylum and genus level. **(a)** Composition heatmap at the phylum level. **(b)** Composition heatmap at the genus level. The colors represent relative abundance standardized by Z-score, with red indicating higher abundance and blue indicating lower abundance. Samples showed substantial interindividual variation in microbial composition, with no clear group-specific clustering between the HDUP and HDNUP groups.

Hierarchical clustering heatmaps at the phylum and genus levels showed substantial interindividual variation in gut microbial composition within both groups. Samples from the HDUP and HDNUP groups were interspersed across the clustering dendrograms and did not form distinct group-specific clusters. Although several taxa varied in relative abundance among individual samples, the overall taxonomic profiles were broadly similar between the two groups (**Figure 4**).

#### 3.3.2 Comparison of taxonomic abundance

**Figure 5:**
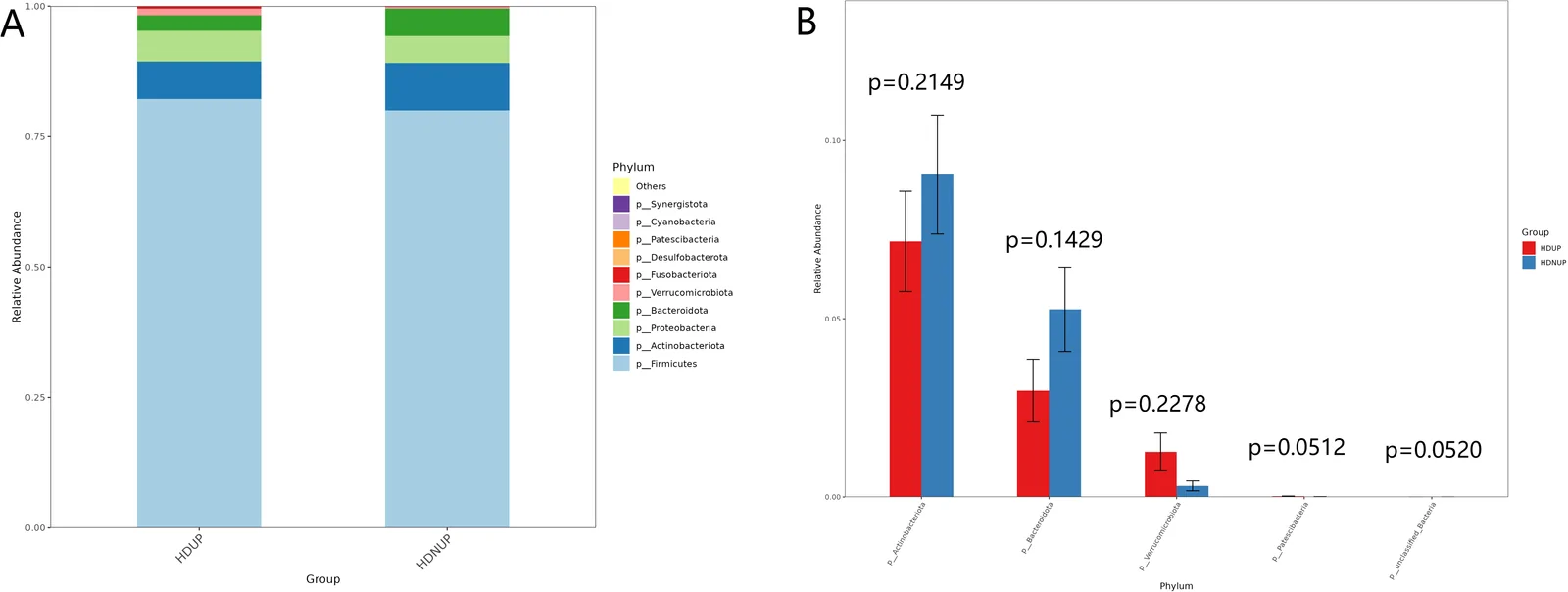
Comparison of gut microbiota composition at the phylum level between HDNUP and HDUP. **(a)** Stacked bar plot of gut microbiota relative abundance at the phylum level in HDNUP and HDUP; **(b)** Comparison of relative abundance of the main dominant phyla, with the corresponding P values labeled in the figure. No statistically significant differences between HDUP and HDNUP (p> 0.05).

**Figure 6:**
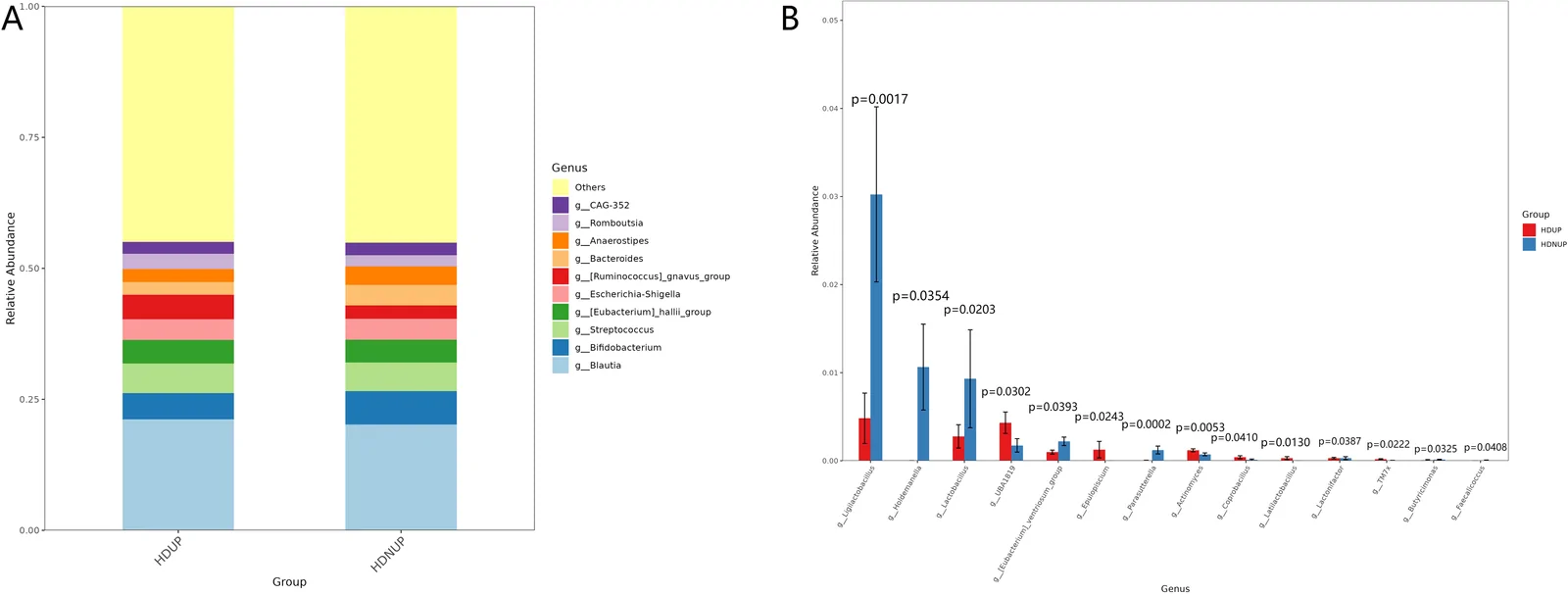
Comparison of gut microbiota composition at the genus level between HDNUP and HDUP. **(a)** Stacked bar plot of gut microbiota relative abundance at the genus level in HDNUP and HDUP; **(b)** Comparison of relative abundance of the main dominant genera, with the corresponding P values labeled in the figure. The Wilcoxon rank-sum test results indicated that 14 bacterial genera exhibited differences in the comparison (p < 0.05). The displayed p values are unadjusted and should be interpreted as exploratory.

At the phylum level, Firmicutes was the predominant phylum in both groups, followed by Actinobacteriota, Proteobacteria, and Bacteroidota. Verrucomicrobiota, Fusobacteriota, Desulfobacterota, Patescibacteria, Cyanobacteria, and Synergistota were present at relatively lower abundances. The overall phylum-level composition was similar between the HDUP and HDNUP groups. Wilcoxon rank-sum tests showed no statistically significant between-group differences in the relative abundances of Actinobacteriota, Bacteroidota, Verrucomicrobiota, Patescibacteria, or unclassified bacteria (all p > 0.05; **Figure 5**). At the genus level, the predominant genera in both groups included Blautia, Bifidobacterium, Streptococcus, [Eubacterium]_hallii_group, Escherichia–Shigella, [Ruminococcus]_gnavus_group, Bacteroides, Anaerostipes, Romboutsia, and CAG-352. The relative contributions of these dominant genera were generally similar between the two groups. In univariable Wilcoxon rank-sum analyses, 14 genera showed nominal between-group differences: Ligilactobacillus, Holdemanella, Lactobacillus, UBA1819, [Eubacterium]_ventriosum_group, Epulopiscium, *Parasutterella*, Actinomyces, Coprobacillus, Latilactobacillus, Lactonifactor, TM7x, Butyricimonas, and Faecalicoccus (all p < 0.05; **Figure 6**). Among these taxa, *Parasutterella* was more abundant in the HDNUP group than in the HDUP group in the unadjusted analysis (p = 0.0002).

Multivariable association analyses were subsequently performed at the phylum, class, order, family, genus, and species levels, with adjustment for age, sex, post-dialysis dry weight, dialysis vintage, the presence of diabetes mellitus, and a documented history of hyperphosphataemia. All six analyses included the same 89 participants.

After Benjamini–Hochberg false-discovery-rate correction, two family-level and two order-level features were significantly associated with pruritus status. At the family level, Actinomycetaceae was positively associated with the HDUP group (coefficient = 1.719, q = 0.0187), whereas Sutterellaceae was inversely associated with the HDUP group (coefficient = −2.085, q = 0.0399). At the order level, Actinomycetales was positively associated with the HDUP group (coefficient = 1.719, q = 0.0158), whereas Burkholderiales was inversely associated with the HDUP group (coefficient = −2.180, q = 0.0173).

No taxon met the false-discovery-rate threshold at the phylum, class, genus, or species level. Parasutterella showed a nominal genus-level association in the adjusted analysis but did not meet the prespecified q < 0.05 threshold. Because Actinomycetaceae is nested within Actinomycetales and Sutterellaceae is nested within Burkholderiales, the significant findings represented two taxonomically nested lineages rather than four independent associations.

#### 3.3.3 LEfSe analysis

LEfSe analysis identified 31 differentially enriched taxonomic features across multiple taxonomic levels using an LDA score threshold of >2.0 and p < 0.05. Fifteen features were enriched in the HDUP group. These primarily included Tyzzerella sp., UBA1819 and its related unclassified species, Epulopiscium and its related unclassified species, Actinomycetales, Actinomycetaceae, Actinomyces, Schaalia odontolytica, Latilactobacillus-related taxa, Coprobacillus-related taxa, and Lactonifactor-related taxa. Sixteen features were enriched in the HDNUP group. These included Ligilactobacillus, Lactobacillus and Lactobacillus salivarius, Holdemanella-related taxa, Bacteroides vulgatus, Bacteroides uniformis, Bacteroides intestinalis, Bifidobacterium bifidum, *Parasutterella* and its related unclassified species, the [Eubacterium] ventriosum group, Sutterellaceae, and Burkholderiales.

**Figure 7:**
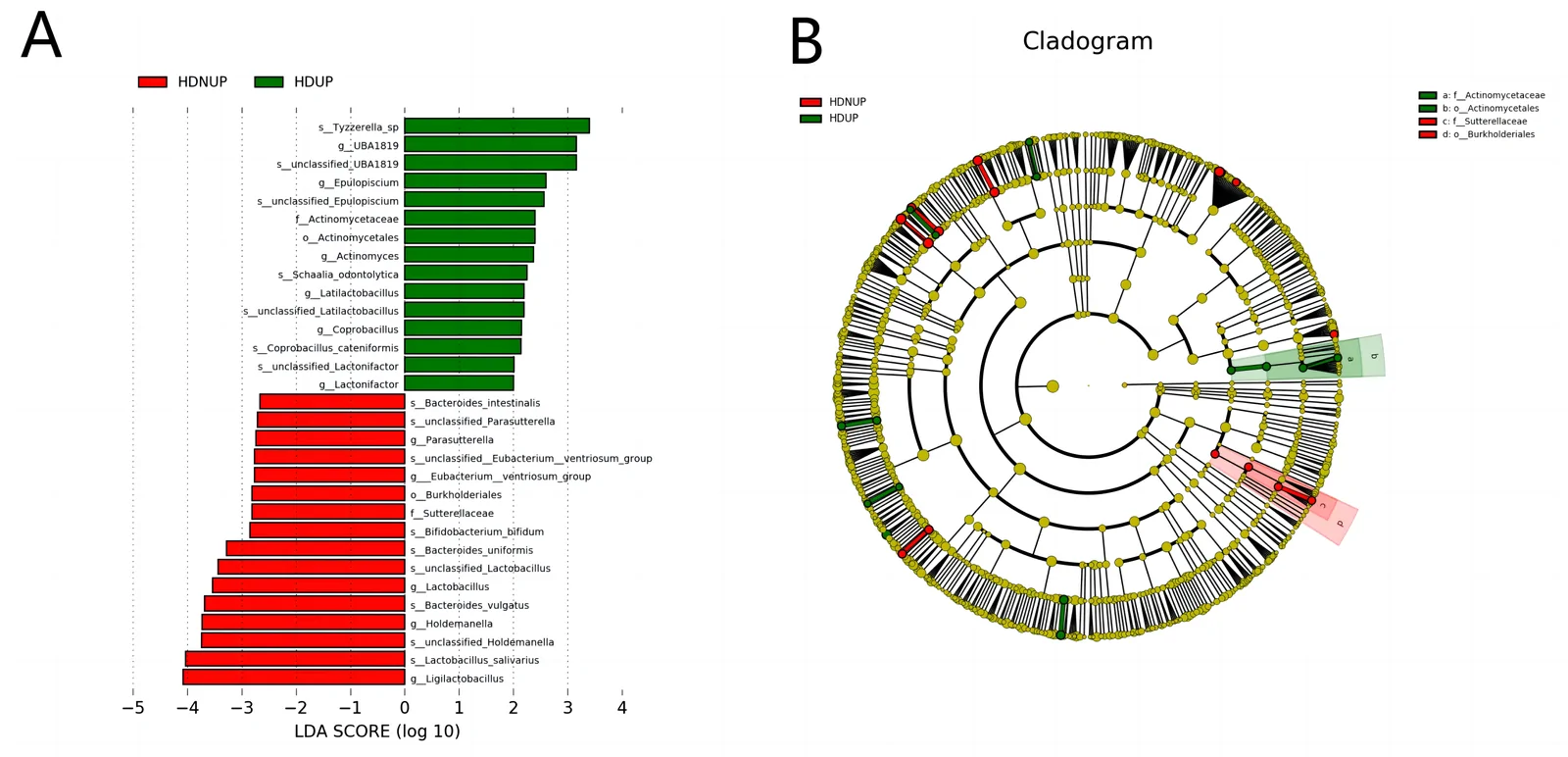
LEfSe analysis. **(a)** Histogram of LDA value distribution for differential taxa identified by LEfSe analysis (LDA> 2); **(b)** Cladogram.The Histogram shows 15 in HDUP and 16 in HDNUP.Cladogram illustrates the taxonomic relationships among the aforementioned divergent taxa.

Actinomycetaceae and Actinomycetales were enriched in HDUP in the LEfSe analysis, whereas Sutterellaceae and Burkholderiales were enriched in HDNUP, consistent with the directions observed in the adjusted MaAsLin2 analysis. Parasutterella was also enriched in HDNUP in the Wilcoxon and LEfSe analyses and showed a nominal association in the adjusted MaAsLin2 analysis in the same direction; however, it did not remain significant after false-discovery-rate correction. Because LEfSe did not incorporate adjustment for clinical covariates or FDR correction, its findings were considered exploratory (**Figure 7**).

### 3.4 Predictive functional analysis

**Figure 8:**
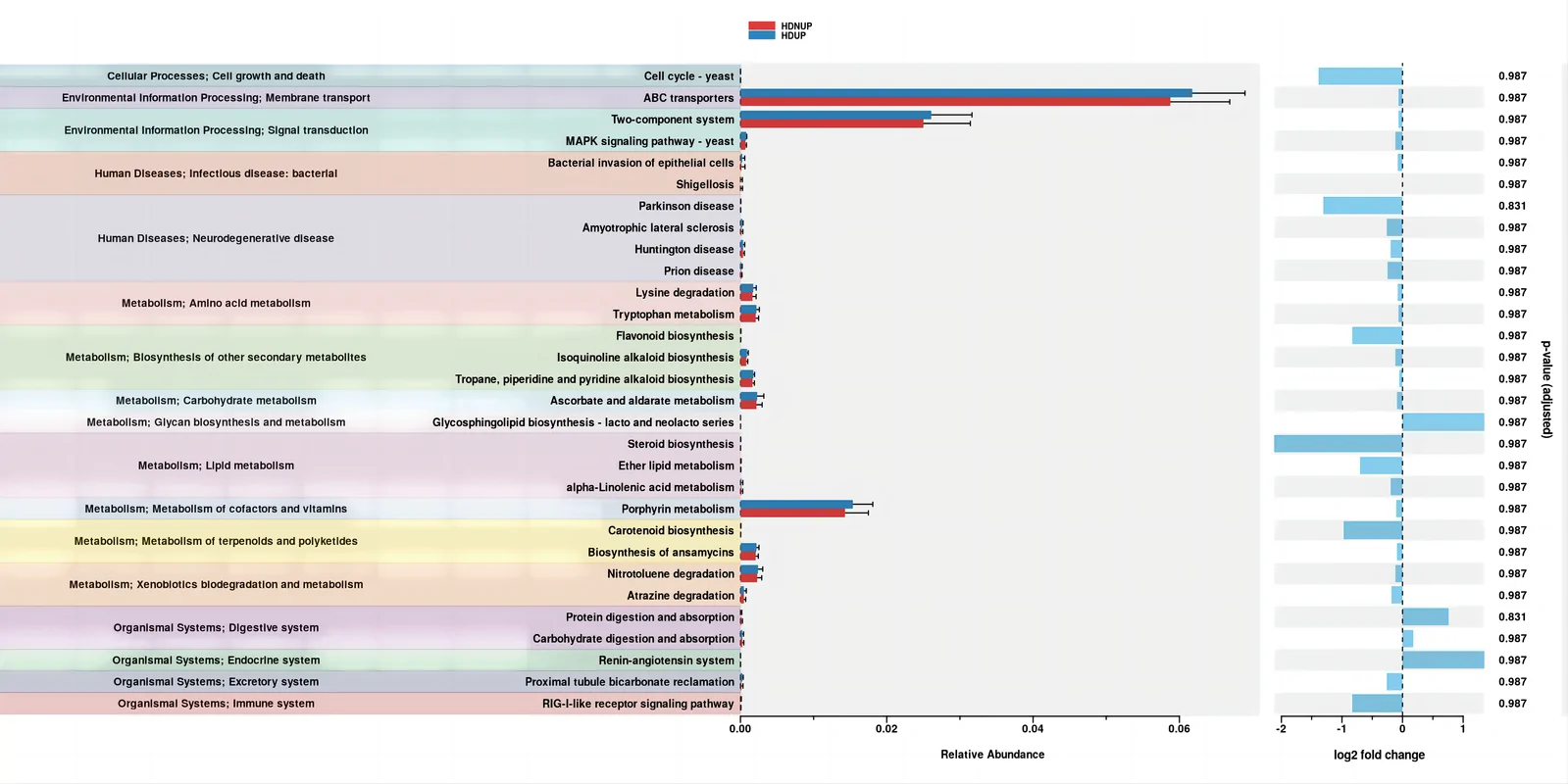
Bar plot of functional abundance difference test results for KEGG pathways. The abscissa represents functional pathways, and the ordinate represents the relative abundance of pathways. HDUP and HDNUP exhibited broadly similar predicted functional profiles, with six pathways showing nominal between-group differences.

PICRUSt2 analysis showed that the overall predicted functional composition was broadly similar between the HDUP and HDNUP groups. Nevertheless, six predicted pathways showed nominal between-group differences. Protein digestion and absorption showed a higher predicted abundance in the HDNUP group (p = 0.0058). In contrast, pathways annotated as Parkinson’s disease (p = 0.0075), carotenoid biosynthesis (p = 0.0171), RIG-I-like receptor signaling (p = 0.0293), flavonoid biosynthesis (p = 0.0351), and ether lipid metabolism (p = 0.0425) showed higher predicted abundances in the HDUP group (**Figure 8**).

## 4 Discussion

The present study did not support broad alterations in gut microbial richness, diversity, or dominant community structure between patients with and without active CKD-aP. Alpha-diversity indices were comparable between groups, and Bray–Curtis-based analysis showed no significant difference in overall community structure. Although unweighted UniFrac analysis detected a statistically significant difference, the effect size was very small (R = 0.03449, p = 0.0183), indicating only subtle variation in the presence or absence of phylogenetic lineages rather than marked community-wide restructuring.

After adjustment, significant associations were confined to two taxonomically nested lineages: the Actinomycetaceae–Actinomycetales lineage was relatively enriched in the HDUP group, whereas the Sutterellaceae–Burkholderiales lineage was relatively enriched in the HDNUP group. No genus- or species-level taxon remained significant after false-discovery-rate correction. These findings suggest that active CKD-aP may be associated with selective lineage-level microbial differences rather than broad community-wide restructuring of the gut microbiota.

This overall pattern was broadly consistent with the findings of Ko et al., who also reported no significant difference in alpha diversity but detected beta-diversity differences between hemodialysis patients with and without uremic pruritus (Ko et al., 2025). However, the specific differential taxa identified in the two studies showed limited overlap. Differences in patient characteristics, diet, medication exposure, bowel habits, pruritus definitions, sequencing platforms, and analytical approaches may partly explain the lack of complete taxonomic concordance (Luo et al., 2021; Du et al., 2024; Lazarevic et al., 2024).

The relationship between uremic solutes and CKD-aP remains uncertain. EUTox-related work has emphasized the complexity of uremic toxicity and the limitations of attributing the uremic syndrome to individual retained solutes (Vanholder et al., 2008). Consistent with this, Bolanos et al. found no individual plasma solute or group of solutes associated with pruritus in patients receiving hemodialysis (Bolanos et al., 2021). In light of these findings, the present microbiome results should not be interpreted as evidence that gut-derived uremic solutes mediate CKD-aP. Rather, our findings indicate subtle and selective differences in microbial relative abundance, the biological and clinical relevance of which remains uncertain.

The Actinomycetaceae–Actinomycetales lineage was positively associated with HDUP in the adjusted MaAsLin2 analysis, with concordant enrichment observed in the exploratory LEfSe analysis. Members of Actinomycetaceae, particularly Actinomyces species, commonly colonize oral and gastrointestinal mucosal sites (Valour et al., 2014; Kononen and Wade, 2015); therefore, their detection in fecal samples may reflect differences in mucosal microbial ecology, including the possibility of oral–gut microbial translocation. However, because oral samples and strain-level tracking data were not available, the anatomical origin of this signal could not be determined. The identical coefficients observed for Actinomycetaceae and Actinomycetales may indicate that the order-level association was largely attributable to the corresponding family-level abundance profile. These findings should therefore be interpreted as one taxonomically nested lineage-level signal rather than two independent associations. The present data cannot establish whether this lineage contributes directly to pruritus or reflects unmeasured host, oral-health-related, or ecological factors.

*Parasutterella* showed a nominal genus-level association in the same direction as the higher-level Sutterellaceae–Burkholderiales signal that remained significant after FDR correction but did not remain significant after multiple-testing correction. Therefore, the present study does not establish *Parasutterella* as an independent or protective microbial factor. Nevertheless, its taxonomic relationship with Sutterellaceae and Burkholderiales provides a rationale for cautious hypothesis generation.

A documented history of hyperphosphataemia was included as a covariate in the expanded MaAsLin2 model. However, contemporaneous serum phosphorus was not separately modeled, and no dedicated stratified or interaction analyses were performed to evaluate the relationship between phosphate status and *Parasutterella* abundance. Therefore, the present study cannot determine whether the nominal *Parasutterella* signal was related to current or cumulative phosphate burden.

Parasutterella has previously been associated with aromatic amino acid and bile acid metabolism (Ju et al., 2019). However, the present study did not measure these metabolites, and a previous metabolomic study in patients receiving hemodialysis did not identify any individual plasma solute or group of solutes associated with pruritus (Bolanos et al., 2021). Therefore, the observed Parasutterella-related signal should not be interpreted as evidence of a gut-derived uremic solute-mediated mechanism. Rather, these metabolic associations provide only biological context for future studies integrating quantitative microbiome analysis with targeted metabolomics.

Exploratory LEfSe analysis also identified several taxa enriched in the HDNUP group, including multiple Lactobacillus-affiliated taxa, Bacteroides spp., Bifidobacterium bifidum, Holdemanella, and the [Eubacterium] ventriosum group. Preclinical studies provide biological context for some of these taxa, although their relevance to CKD-aP remains uncertain. Lactobacillus salivarius BP121 has been reported to reduce selected circulating uremic solutes, including indoxyl sulfate and p-cresyl sulfate, and to alleviate intestinal barrier injury and inflammation in a cisplatin-induced kidney injury model (Lee et al., 2020). Human gut-associated Bifidobacterium species, including B. bifidum, can convert exogenous indole into indole-3-lactic acid, suggesting a potential role in modulating indole-related microbial metabolism (Yong et al., 2024; Konishi et al., 2025). Holdemanella biformis has also been linked to short-chain fatty acid production and regulation of GLP-1 signaling in obese mice (Romaní-Pérez et al., 2021). Nevertheless, these effects are species- or strain-dependent and have mainly been demonstrated in experimental models unrelated to CKD-aP. Accordingly, these LEfSe-enriched taxa cannot be classified as protective solely on the basis of 16S rRNA gene-based relative-abundance data.

Interpretation of Bacteroides-related findings also requires caution, because distinct species and strains within this genus may exert differing effects on carbohydrate fermentation, propionate production, mucosal homeostasis, and inflammatory responses. By contrast, exploratory LEfSe analysis identified enrichment of Tyzzerella-related taxa, UBA1819, Epulopiscium, Actinomyces-related lineages, Schaalia odontolytica, Latilactobacillus, Coprobacillus, and Lactonifactor-related taxa in HDUP. Tyzzerella nexilis encodes enzymes capable of degrading O-linked glycans, including mucin-derived substrates (Kulinich et al., 2020), however, this metabolic capability should not be interpreted as evidence of mucosal injury. Actinomyces and Schaalia are commonly found in the oral microbiota. Experimental studies have shown that ectopic intestinal colonization by certain oral bacteria can induce Th1-associated inflammatory responses in susceptible hosts (Atarashi et al., 2017). Nevertheless, the fecal detection of Actinomyces- and Schaalia-related taxa in the present study may only reflect differences in oral–gut microbial ecology, and their anatomical origin and functional relevance cannot be determined without paired oral and fecal sampling, strain-level analysis, and assessment of intestinal barrier function. The biological roles of UBA1819 and Epulopiscium remain insufficiently characterized; therefore, they should be regarded as exploratory microbial signatures rather than pathogenic taxa.

It should be emphasized that the 16S rRNA sequencing data used in this study were compositional and reflected relative rather than absolute microbial abundance. Therefore, the nominally higher relative abundance of Parasutterella in HDNUP may have resulted from an increase in its absolute abundance, a reduction in other taxa, or a combination of both. The present data cannot distinguish among these possibilities. Absolute quantification using *Parasutterella*-targeted qPCR, digital PCR, spike-in normalization, or quantitative metagenomic approaches will be required to validate whether the observed relative-abundance difference reflects a genuine change in bacterial load.

PICRUSt2 predicted a higher abundance of protein digestion and absorption in HDNUP and of pathways annotated as RIG-I-like receptor signaling, ether lipid metabolism, carotenoid biosynthesis, flavonoid biosynthesis, and Parkinson’s disease in HDUP. These findings do not demonstrate actual microbial metabolic activity or host pathway activation. PICRUSt2 infers potential gene content from 16S phylogeny, cannot fully resolve strain-level variation, and does not determine whether genes are expressed (Douglas et al., 2020). Thus, the RIG-I-like receptor signaling annotation does not demonstrate host antiviral pathway activation, and the Parkinson’s disease annotation does not indicate neurodegeneration. These predicted functional differences should be regarded as hypothesis-generating and require direct evaluation using shotgun metagenomics, metatranscriptomics, metabolomics, and host biomarker measurements. Together with the taxonomic findings and previous experimental evidence, they also support further investigation of lipid metabolism, redox processes, immune-active microbial products, bile acid profiles, and aromatic amino acid-derived metabolites.

Although microbiota-modulating interventions have been investigated in patients with CKD for their effects on microbial composition, inflammatory markers, and selected retained solutes, whether such interventions improve CKD-aP remains unknown. Dietary fiber, prebiotics, probiotics, and synbiotics have been evaluated in patients with CKD, including those receiving hemodialysis. A recent network meta-analysis comprising 53 studies suggested that multi-strain probiotic preparations, particularly combinations containing three or more strains, may improve selected renal, metabolic, inflammatory, and oxidative-stress-related outcomes (Li et al., 2025). In a randomized trial involving patients receiving hemodialysis, probiotic supplementation modified aspects of gut microbial composition and reduced several uremic retention solutes (Liu et al., 2020). Meta-analytic evidence also suggests that probiotic, prebiotic, and synbiotic interventions may reduce circulating p-cresyl sulfate, endotoxin, and selected inflammatory and oxidative-stress markers (Nguyen et al., 2021). However, these studies did not specifically evaluate CKD-aP, and improvements in biochemical surrogate outcomes cannot be assumed to translate into clinically meaningful relief of pruritus. Therefore, microbiota modulation should currently be regarded as an investigational therapeutic strategy rather than an established treatment for CKD-aP. Future randomized trials should incorporate validated pruritus outcomes, such as the Worst Itching Intensity Numerical Rating Scale (WI-NRS) or the 5-D Itch Scale, together with measures of sleep disturbance and quality of life, absolute microbial quantification, and targeted metabolomic and inflammatory profiling.

This study has several limitations. First, its cross-sectional design precludes determination of temporal direction or causality, and the single-center setting may limit the generalizability of the findings. Because only a subset of patients treated at the study center was approached for eligibility assessment and some clinically eligible patients did not provide qualified fecal samples, selection bias cannot be completely excluded. Second, pruritus was analyzed primarily as a binary phenotype according to the presence or absence of active symptoms. Therefore, the study could not determine whether microbial features varied with symptom severity or showed a dose–response relationship with pruritus intensity. Third, although the serum phosphorus concentration measured closest to fecal sampling did not differ significantly between the groups, a documented history of hyperphosphataemia was more frequent in the HDUP group than in the HDNUP group (66.7% vs. 45.5%, p = 0.044). These variables reflected different clinical time frames: the documented history represented prior clinical information, whereas the laboratory value represented phosphate status at a single time point. Although documented hyperphosphataemia was included in the expanded MaAsLin2 model, it did not fully capture the duration, severity, or cumulative burden of phosphate abnormalities. Repeated serum phosphorus and intact parathyroid hormone measurements and cumulative CKD–mineral and bone disorder burden were not available.

More broadly, although the expanded MaAsLin2 model adjusted for age, sex, post-dialysis dry weight, dialysis vintage, the presence of diabetes mellitus, and a documented history of hyperphosphataemia, residual confounding cannot be excluded. Several potentially relevant factors, including dietary intake, constipation, stool consistency, intestinal transit time, oral health, xerosis, antipruritic treatment, phosphate-binder exposure, iron preparations, and other medications, were not collected prospectively in a sufficiently standardized and complete manner. The modest sample size also limited the number of covariates that could be included without compromising model stability. Accordingly, the identified lineage-level associations should be interpreted as selected-covariate-adjusted exploratory signals rather than independent or causal microbial effects.

In addition, 16S rRNA gene sequencing provides limited species- and strain-level resolution and generates compositional relative-abundance data rather than absolute bacterial loads. Consequently, the nominal relative-abundance pattern involving *Parasutterella* in HDNUP cannot be interpreted as evidence of an absolute increase in this genus. Targeted qPCR, digital PCR, spike-in normalization, or other quantitative microbiome approaches are required for validation. Finally, the limited sample size precluded reliable subgroup and interaction analyses according to clinically relevant variables; therefore, potential effect modification across patient subgroups could not be assessed. Future adequately powered prospective multicenter studies should incorporate standardized assessment of CKD–mineral and bone disorder-related factors, dietary and gastrointestinal variables, medication exposure, quantitative microbial abundance, targeted metabolomics, and repeated evaluation of pruritus severity.

## 5 Conclusions

In this cross-sectional study of patients receiving maintenance hemodialysis, active CKD-associated pruritus was associated with selective differences in gut microbial relative abundance rather than broad alterations in microbial richness, diversity, or dominant community structure. After adjustment for age, sex, post-dialysis dry weight, dialysis vintage, the presence of diabetes mellitus, and a documented history of hyperphosphataemia, the Actinomycetaceae–Actinomycetales lineage was relatively enriched in patients with active CKD-aP, whereas the Sutterellaceae–Burkholderiales lineage was relatively enriched in patients without active pruritus. No genus- or species-level taxon remained statistically significant after FDR correction. *Parasutterella* showed a nominal genus-level association in a direction consistent with the broader Sutterellaceae–Burkholderiales signal but should not be interpreted as an independent or protective microbial factor. These exploratory findings require validation in prospective multicenter studies incorporating absolute microbial quantification, targeted metabolomic profiling, and repeated assessment of pruritus severity.

## Data Availability

The datasets presented in this study can be found in online repositories. The names of the repository/repositories and accession number(s) can be found below: https://www.ncbi.nlm.nih.gov/, PRJNA1480656.
